# Impact of Cribriform Pattern on Progression-Free Survival After Radical Prostatectomy in Gleason Score 8-10 Prostate Cancer

**DOI:** 10.5152/eurasianjmed.2025.25804

**Published:** 2025-04-21

**Authors:** Tyler Walburn, Yetkin Tuaç, Çağdaş Aktan, Okan Argun, Luke W. Chen, David D. Yang, Shalini Moningi, Jonathan E. Leeman, Peter F. Orio, Paul L. Nguyen, Anthony V. D’Amico, Mutlay Sayan

**Affiliations:** 1 Department of Radiation Oncology, Brigham and Women’s Hospital and Dana Farber Cancer Institute, Harvard Medical School, Boston, MA, USA; 2 Department of Statistics, Ankara University Faculty of Science, Ankara, Türkiye; 3 Department of Medical Biology, Bandırma Onyedi Eylül University Faculty of Medicine, Balıkesir, Türkiye

**Keywords:** Cribriform pattern, progression-free survival, prostate cancer, radical prostatectomy

## Abstract

**Background:**

Although extensive research highlights the detrimental effect of cribriform pattern 4 (CP4) on survival in non-high-risk prostate cancer (PC), its prognostic significance in high-risk PC is not well understood.

**Methods:**

The individual patient data from the The Cancer Genome Atlas (TCGA) database following radical prostatectomy was retrospectively examined. A predefined statistical analysis was conducted to evaluate the potential association between CP4 and progression-free survival (PFS).

**Results:**

Of the 135 patients examined, CP4 was present in 66 (48.9%). Median follow-up was 45.93 months (interquartile range: 22.87, 80.10). Cribriform pattern 4 was associated with a significantly reduced PFS (subdistribution hazard ratio, 1.99; 95% CI, 1.01-3.92; *P* = .045) following adjustment for covariates.

**Conclusions:**

The presence of CP4 in high-risk Gleason 8-10 PC portends worse PFS. Further studies are warranted to fully understand its implications in risk stratification and post-operative management of PC.

**Main Points**

- Cribriform pattern presence is linked to poorer progression-free survival in patients with Gleason score 8-10 prostate cancer.
- Among 135 patients, cribriform pattern was present in 48.9% of cases and was independently linked to an almost twofold higher risk of disease progression
- These findings suggest that the cribriform pattern may serve as a valuable histopathological marker to refine risk stratification in high-risk prostate cancer.
- Additional studies are necessary to assess the impact of the cribriform pattern on postoperative management and treatment escalation decisions.

## Introduction

Prostate cancer (PC) diagnosis and prognosis rely heavily on histopathological examination, with the Gleason scoring system serving as the cornerstone for tumor grading.^1^^-^^5^ Recent advancements in the understanding of the histopathologic underpinnings of PC have highlighted the significance of specific architectural patterns within Gleason grade groups, particularly cribriform pattern 4 (CP4). There is growing evidence demonstrating the clinical impact CP4 has in relation to worse clinical outcomes, including prostate-specific antigen (PSA) failure, recurrence-free survival, distant metastases, and prostate cancer-related mortality.^6^^-^^15^ However, the existing data are predominantly in non-high risk Gleason grade PC, and only 1 study has examined CP4 in Gleason 8 disease.^7^ Thus, the precise impact of CP4 on disease progression and survival outcomes in high-grade PC, specifically within the Gleason 8-10 cohort, remains incompletely understood.

This study aimed to fill this knowledge gap by analyzing the clinical data from the TCGA database to evaluate the influence of CP4 on disease progression in patients with Gleason 8-10 prostate cancer, accounting for established prognostic factors. The aim was to provide evidence for the prognostic implications of CP4 in this high-risk population to offer valuable insights into risk stratification and support further investigation of CP4 in prospective adjuvant treatment studies.

## Materials and Methods

### Baseline Patient Characteristics

Patient information extracted from the TCGA database was retrospectively analyzed in this study. Collected data included clinical variables such as age, pathological (p) tumor (T) stage, margin status, pre-radical prostatectomy PSA values, and the presence of CP4. Follow-up duration was measured from the date of surgery to the most recent follow-up or recorded death. This study, which involves human participants, was completed following the guidelines of the Declaration of Helsinki. As it utilizes the publicly available and de-identified TCGA database, it met the exception criteria for Institutional Review Board review at Ethics committee of Dana-Farber Cancer Institute (Exception no.: 491519; Date: 02/02/2024), and written informed consent for participation was waived.

### Statistical Methods

#### Clinical Factor Distribution Based on Cribriform Pattern 4 Status

A predefined statistical analysis plan was followed. Descriptive statistics summarized the clinical characteristics of 135 prostate cancer patients with a Gleason score of 8-10, stratified based on CP4 presence or absence. Categorical variables were compared using Pearson’s chi-squared test, while the Wilcoxon 2-sample test assessed differences in continuous variables.^16^ The reverse Kaplan–Meier method was used to evaluate the distribution of follow-up times, while differences were assessed using the log-rank test.^17^

### Adjusted Hazard Ratios for Progression-Free Survival

Progression-free survival was the primary endpoint, measured from the time of radical prostatectomy (RP) to the earliest occurrence of disease progression or death.^18^^-^^20^ The Fine-and-Gray model was used for both uni- and multi-variable competing risk regression analyses to evaluate the relationship between CP4 presence and progression-free survival (PFS).^21^ Time 0 was set as the date of RP. The model included covariates such as age, pre-radical prostatectomy PSA level, pT stage, Gleason scores, and the status of the surgical margins. Subdistribution hazard ratios (SDHRs) and 95% CIs with *P*-values were reported for these covariates.

### Estimated Progression-Free Survival After Covariate Adjustment

To illustrate the findings, Kaplan–Meier estimates of PFS were generated, adjusting for covariates and stratifying by CP4 presence or absence.^22^ These estimates of PFS, along with their corresponding 95% CIs, were adjusted for age, pre-radical prostatectomy PSA level, pT stage, Gleason scores, and the status of the surgical margins. The statistical significance of the adjusted Kaplan–Meier plots was evaluated using the log-rank test. A *P*-value of <.05 was considered the threshold for statistical significance in all analyses. All statistical analyses were completed using R software (v4.2.3).

## Results

### Clinical Factor Distribution by Cribriform Pattern 4 Status

Of the 135 patients included in the analysis, 66 (48.89%) exhibited CP4. Table 1 presents their characteristics, categorized based on CP4 presence. Patients with CP4 had a longer median follow-up compared to those without CP4 [52.53 months (interquartile range [IQR]: 22.67-70.23) vs. 40.73 months (IQR: 25.63-80.10), *P* = .043]. Baseline PSA [9.25 ng/mL (IQR: 5.82, 15.1) versus 8.10 ng/mL (IQR: 5.08, 11.8), *P* = .276], the rates of positive prostatectomy margins (41% vs. 54%, *P* = .140), and ≥T3a stage (84% versus 82%, *P* = .800) were similar between the 2 groups.

### Adjusted Hazard Ratios for Progression-Free Survival

Over a median follow-up period of 45.93 months (IQR: 22.87, 80.10), 46 (34.07%) patients had biochemical recurrence, 5 (3.70%) patients had locoregional recurrence, 1 (0.74%) patient had distant metastasis, 1 (0.74%) patient had a new primary tumor, and 1 (0.74%) patient died. As shown in Table 2, CP4 presence was independently linked to a significantly shorter PFS (SDHR, 1.99; 95% CI, 1.01-3.92; *P* = .045) after adjusting for established covariates.

### Adjusted Estimates of Progression-Free Survival

**Figure 1.**
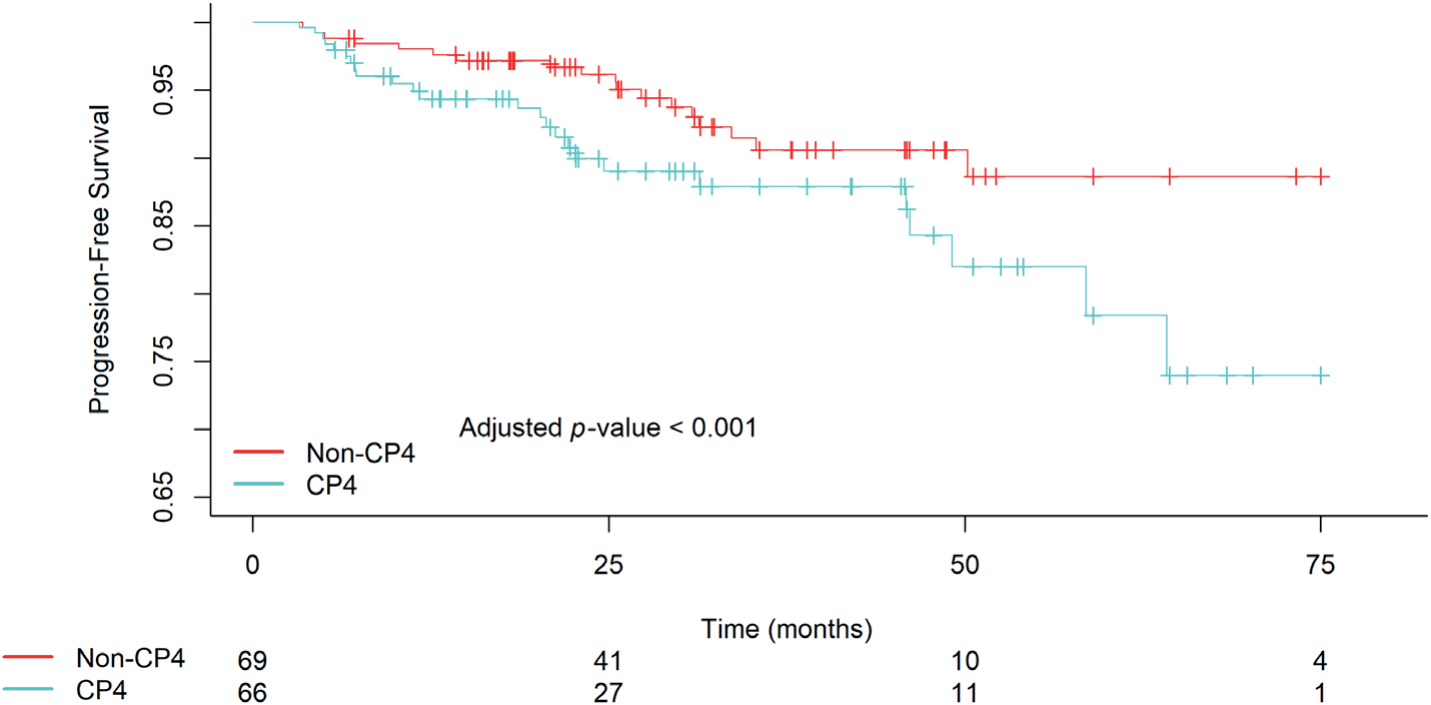
Covariate adjusted estimates of PFS by the cribriform pattern 4 status. CP4, cribriform pattern 4; non-CP4, non-cribriform pattern 4, PFS, progression-free survival.

Figure 1 illustrates the adjusted PFS estimates stratified by CP4 status. Patients with CP4 exhibited significantly lower adjusted PFS compared to those without CP4 (*P* < .001). The adjusted 3-year PFS rate was 88.31% (95% CI, 73.00%-100.00%) in patients with CP4, whereas it was 90.76% (95% CI, 77.84%-100.00%) in those without CP4.

## Discussion

After adjusting for known prognostic factors, the analysis revealed that the presence of CP4 in Gleason 8-10 PC is significantly associated with lower PFS. This finding is clinically meaningful as it aids in the understanding of the relevant pathologic features involved in the risk-stratification of high-risk PC and supports the study of CP4 in prospective randomized trials to better understand its clinical implications and role in risk-stratification.

Currently, the standard of care for men with high-risk PC is either RP with pelvic lymph node dissection (PLND) or radiation therapy with androgen deprivation therapy (ADT; abiraterone is added for selected very-high-risk PC).^23^ In the setting of PSA persistence or recurrence following RP/PLND, salvage radiotherapy with or without ADT has been widely adopted after 3 clinical trials failed to demonstrate a benefit for adjuvant treatment over salvage therapy in all-comers.^24^^-27^ Early salvage (initiation of therapy once PSA has reached 0.1 ng/mL) has been widely adopted after a multi-institutional study demonstrated a distant metastasis benefit.^28^ However, the door has not been shut entirely on adjuvant treatment. One large multinational study identified a high-risk subset of patients, specifically Gleason 8-10 and pT3-4 disease, that have an all-cause mortality benefit with adjuvant treatment compared to early salvage;^29^ thus, there is now a population in which adjuvant therapy appears reasonable.

While the timing of postoperative therapy is clearly critical, the duration of ADT seems to play a more complex role. The Radiotherapy and Androgen Deprivation In Combination After Local Surgery–Hormone Duration (RADICALS-HD) trial concluded that among patients receiving postoperative radiotherapy and considered suitable for no, short-term, or long-term ADT, there was no improvement in outcomes with the addition of ADT in the overall study population.^30^ However, it is important to note that only 72 patients (14.6%) in the trial had Gleason score 8-10 disease, limiting the ability to assess the potential benefits of prolonged ADT in this higher-risk subgroup. The findings suggest that men with high-risk features, such as Gleason score 8-10 disease and the presence of CP4, may represent a distinct population that could benefit from intensified or prolonged therapies. This highlights the need for post-randomization analysis of the RADICALS-HD trial to evaluate the role of ADT in very-high-risk patients, including those with pathologic features like CP4, to refine postoperative treatment strategies further.

The findings findings contribute to the growing understanding of the clinical relevance of CP4. To date, its presence appears to portend worse PFS and mortality in predominantly non-high-risk PC. Its growing clinical relevance has been acknowledged by the International Society of Urological Pathology Consensus Conference on Gleason Grading, which now upgrade patients to Gleason pattern 4 if this architectural pattern is present.^2^ One study has examined CP4 in Gleason 8 PC and found an association with worse biochemical recurrence-free survival and metastasis free survival (MFS) on multivariate analysis (MVA).^7^ Importantly this study did not include Gleason 9 or 10 disease, and the findings address this knowledge gap in the data and add to the current understanding of CP4 in high-risk PC.

Several points require further discussion. First, the retrospective nature of this study creates limitations in drawing concrete conclusions. With this in mind, the results appear congruent with much of the existing data. Second, the follow-up period was longer in patients with CP4, which allowed more time to detect failures and could have biased results. Despite this, CP4 was associated with PFS on MVA, which accounted for median follow-up time. Third, the lack of information regarding adjuvant treatments within the dataset poses a limitation, possibly affecting the interpretation of the PFS data. Finally, one could argue that PFS is not a clinically meaningful endpoint as it includes biochemical recurrence, which does not correlate with MFS or overall survival.^31^ While this limitation is acknowledged, the median follow-up for the study subjects was not long enough to detect a meaningful difference in either of these alternative endpoints. However, having detected a PFS difference within the short follow-up period demonstrates the potential impact CP4 may have on prognosis.

The presence of CP4 is associated with reduced PFS in patients with Gleason 8-10 PC and warrants further study in prospective trials to better inform risk stratification in high-risk PC in the post-operative setting.

## Figures and Tables

**Table 1.** Clinical and Treatment Factor Distribution by Cribriform Pattern 4 Status

	CP4 (n = 66)	Non-CP4 (n = 69)	*P*
Age, years, median (IQR)	63 (57, 67)	62 (57, 66)	.497
Baseline PSA, ng/mL, median (IQR)	9.25 (5.82, 15.1)	8.10 (5.08, 11.8)	.276
Prostatectomy tumor stage, No. (%)			.800
T2	10 (16%)	12 (18%)	
T3a or higher	54 (84%)	56 (82%)	
Prostatectomy Gleason score, No. (%)			.800
8	28 (42%)	28 (41%)	
9-10	38 (58%)	41 (59%)	
Prostatectomy margin status, No. (%)			.140
Negative	39 (59%)	30 (46%)	
Positive	27 (41%)	35 (54%)	
Prostatectomy nodal status, No. (%)			.400
Negative (N0)	47 (75%)	44 (68%)	
Positive (N1)	16 (25%)	21 (32%)	
Follow-up (month), median (IQR)	52.53 (22.67, 70.23)	40.73 (25.63, 80.10)	.043

CP4, cribriform pattern 4; IQR, interquartile range; non-CP4, non-cribriform pattern 4; PSA, prostate-specific antigen.

**Table 2.** Adjusted Hazard Ratios for Progression-Free Survival

Covariates	Univariable			Multivariable		
	HR	95% CI	*P *	SDHR	95% CI	*P*
Age (years)	0.970	0.938-1.004	.088	0.963	0.929-0.999	.047
Baseline PSA, ng/mL						
<4	0.874	0.306-2.498	.802	0.495	0.151-1.622	.246
4-10	Reference	Reference		Reference	Reference	
>10	0.789	0.437-1.426	.433	0.390	0.184-0.827	.014
Prostatectomy tumor stage						
T2	Reference	Reference		Reference	Reference	
T3a or higher	4.346	0.985-19.17	.052	4.202	0.853-20.69	.077
Prostatectomy Gleason score						
8	Reference	Reference		Reference	Reference	
9-10	1.682	0.899-3.146	.104	1.983	0.941-4.177	.071
Prostatectomy margin status						
Negative	Reference	Reference		Reference	Reference	
Positive	0.929	0.519-1.666	.807	0.796	0.399-1.589	.518
Cribriform pattern 4						
Non-CP4	Reference	Reference		Reference	Reference	
CP4	1.534	0.870-2.701	.139	1.994	1.013-3.924	.045

CP4, cribriform pattern 4; HR, hazard ratio; non-CP4, non-cribriform pattern 4; PSA, prostate-specific antigen; SDHR, subdistribution hazard ratio.

## Data Availability

The data that support the findings of this study are available on request from the corresponding author.
